# AMN107 (nilotinib): a novel and selective inhibitor of *BCR-ABL*

**DOI:** 10.1038/sj.bjc.6603170

**Published:** 2006-05-23

**Authors:** E Weisberg, P Manley, J Mestan, S Cowan-Jacob, A Ray, J D Griffin

**Affiliations:** 1 Department of Adult Oncology, Dana Farber Cancer Institute, 44 Binney Street, Boston, MA 02115, USA; 2 Novartis Institutes of Biomedical Research, Basel, Switzerland

**Keywords:** *BCR-ABL*, AMN107, nilotinib, dasatinib, imatinib-resistance

## Abstract

Chronic myelogenous leukaemia (CML) and Philadelphia chromosome positive (Ph+) acute lymphoblastic leukaemia (ALL) are caused by the *BCR-ABL* oncogene. Imatinib inhibits the tyrosine kinase activity of the *BCR-ABL* protein and is an effective, frontline therapy for chronic-phase CML. However, accelerated or blast-crisis phase CML patients and Ph+ ALL patients often relapse due to drug resistance resulting from the emergence of imatinib-resistant point mutations within the *BCR-ABL* tyrosine kinase domain. This has stimulated the development of new kinase inhibitors that are able to over-ride resistance to imatinib. The novel, selective *BCR-ABL* inhibitor, AMN107, was designed to fit into the ATP-binding site of the *BCR-ABL* protein with higher affinity than imatinib. In addition to being more potent than imatinib (IC50<30 nM) against wild-type *BCR-ABL*, AMN107 is also significantly active against 32/33 imatinib-resistant *BCR-ABL* mutants. In preclinical studies, AMN107 demonstrated activity *in vitro* and *in vivo* against wild-type and imatinib-resistant *BCR-ABL*-expressing cells. In phase I/II clinical trials, AMN107 has produced haematological and cytogenetic responses in CML patients, who either did not initially respond to imatinib or developed imatinib resistance. Dasatinib (BMS-354825), which inhibits Abl and Src family kinases, is another promising new clinical candidate for CML that has shown good efficacy in CML patients. In this review, the early characterisation and development of AMN107 is discussed, as is the current status of AMN107 in clinical trials for imatinib-resistant CML and Ph+ ALL. Future trends investigating prediction of mechanisms of resistance to AMN107, and how and where AMN107 is expected to fit into the overall picture for treatment of early-phase CML and imatinib-refractory and late-stage disease are discussed.

Chronic myelogenous leukaemia (CML) constitutes 15% of adult leukaemias, with approximately 4600 newly diagnosed cases per annum in the United States. The initial, chronic phase of the disease has a median duration of 4–6 years and is characterised by overproduction of immature myeloid cells and mature granulocytes in the spleen, bone marrow, and peripheral blood. Without therapeutic intervention, after a mean latency period of 4–6 years, the disease progresses via an accelerated phase, marked by the presence of primitive blast cells in the bone marrow and peripheral blood, and finally advances to the ‘blast-crisis’ phase, characterised by over 30% undifferentiated blasts in the bone marrow and peripheral blood, and for which median survival is 18 weeks (Kantarjian and Talpaz, 1988).

The *BCR-ABL* oncogene, which results from a reciprocal t(9;22) chromosomal translocation, encodes a chimeric *BCR-ABL* protein having constitutively activated Abl tyrosine kinase activity, and is the underlying cause of CML (Bartram *et al*, 1983; Groffen *et al*, 1984; Lugo *et al*, 1990). The 210 kDa *BCR-ABL* protein is expressed in CML patients, whereas a 190 kDa *BCR-ABL* protein, resulting from an alternative breakpoint in the *BCR* gene, is expressed in Ph+ acute lymphoblastic leukaemia (ALL) patients (Chan *et al*, 1987).

The discovery that CML is due to the activity of *BCR-ABL* prompted the design and development Novartis Pharma AG, WKL-136.7.86, Klybeckstrasse 141, CH-4057 Basel, Switzerland of imatinib (Glivec®, Gleevec™, STI571; Novartis Pharma AG), a small molecule kinase inhibitor that targets the PDGFR, c-Kit and Abl kinases (Druker *et al*, 1996; Buchdunger *et al*, 2000). Imatinib provides an effective and durable therapy for CML, inducing complete haematologic remissions (normal leucocyte count in peripheral blood) in the majority (98%) of newly diagnosed patients in the chronic phase of the disease, and complete cytogenetic responses (no detectable Ph+ cells from ⩾20 bone marrow cells in metaphase) in a high percentage (86%) of patients (Simonsson, 2005). Primary resistance to imatinib only occurs occasionally in chronic-phase CML patients, and recent analysis of the IRIS study shows a low and decreasing annual rate of progression (resulting in death) after 1, 2, 3 and 4 years of therapy of 3.4, 7.5, 4.8 and 1.5%, respectively, possibly as a result of patients with the worse prognosis progressing relatively early. In 39% of newly diagnosed chronic-phase CML patients, therapy with a standard dose of imatinib for 12 months leads to a major molecular response comprising of 1000-fold reduction in *BCR-ABL* transcript levels, which is associated with a reduced risk of disease progression (Hughes *et al*, 2003). However, advanced (accelerated or blast crisis) phase CML and Ph+ ALL patients show significantly decreased response rates to treatment with imatinib monotherapy, with relapse common within a year (Ottmann *et al*, 2002; Sawyers *et al*, 2002); acquired resistance is less commonly observed in the case of newly diagnosed Ph+ ALL patients receiving combination therapy with chemotherapy.

Resistance frequently results from the emergence of point mutations within the kinase domain of the *BCR-ABL* protein that reduce the binding affinity of imatinib, although it is occasionally associated with amplification of the *BCR-ABL* gene (Gorre *et al*, 2001). Most mutations that confer resistance to imatinib are distributed throughout the Abl kinase domain. However, the most resistant ones, such as many of those found in the P-loop, often occur at or near residues that are in direct contact with the drug. The degree of resistance ranges from a few fold for some of the A-loop mutants, up to complete resistance for the T315I mutation, which precludes imatinib from binding. Overall, the steady rate of developing resistance to imatinib has suggested that new kinase inhibitors could be of clinical value, particularly if they could override imatinib resistance and bind with higher affinity to *BCR-ABL*.

## AMN107 (NILOTINIB)

**Figure 1:**
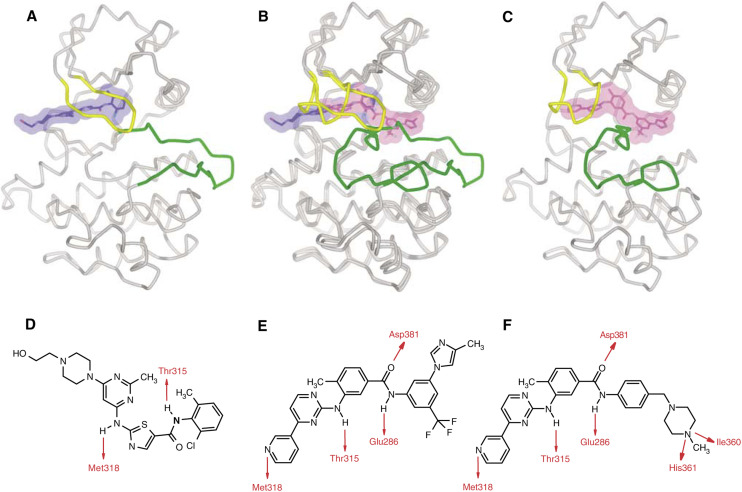
Structures of Abl kinase (**A**) in the active (Fendrich *et al*, 2006) and (**C**) inactive states, with dasatinib (blue) docked and nilotinib (magenta) as bound in the crystal structure (Weisberg *et al*, 2005), respectively. The differing conformations of the glycine-rich or P-loop (yellow) and the activation loop (green) are induced or stabilised by the different binding modes of the two inhibitors. (**B**) shows a superposition of the two distinct conformations, emphasising how dasatinib and nilotinib occupy different parts of the cleft between the N- (upper) and C-terminal (lower) lobes of the kinase. The corresponding aspects of the molecular structures of (**D**) dasatinib and (**E**) nilotinib are depicted, with their respective H-bond interactions with the Abl kinase domain indicated in red, in comparison to imatinib (**F**).

Rational design of novel inhibitors exhibiting effectiveness against imatinib-resistant mutants of *BCR-ABL* was carried out by researchers at Novartis Pharmaceuticals, based upon the crystal structure of the imatinib-Abl complex (Schindler *et al*, 2000; Nagar *et al*, 2002; Manley *et al*, 2004). It was hypothesised that the potency and selectivity of imatinib (Figure 1F) could be improved by maintaining binding to the inactive conformation of the Abl kinase domain, but incorporating alternative binding groups to the *N*-methylpiperazine moiety, while preserving an amide pharmacophore to retain H-bond interactions to Glu286 and Asp381. This led to the development of AMN107 (nilotinib; Figure 1E), a high-affinity aminopyrimidine-based ATP-competitive inhibitor that decreases proliferation and viability of wild-type *BCR-ABL*- and imatinib-resistant *BCR-ABL* mutant-expressing cells *in vitro* by selectively inhibiting *BCR-ABL* autophosphorylation (Table 1). AMN107 exhibits superior potency to imatinib as an inhibitor of wild-type *BCR-ABL* in a wide range of CML-derived and transfected cell lines (Golemovic *et al*, 2005; Weisberg *et al*, 2005). This *in vitro* profile translates into *in vivo* efficacy, where AMN107 has been shown to prolong the survival of mice injected with *BCR-ABL*-transformed haematopoietic cell lines or primary marrow cells, and to prolong survival in imatinib-resistant CML mouse models (Weisberg *et al*, 2005).

As well as being designed to bind more tightly to the *BCR-ABL* protein in an attempt to enhance efficacy, AMN107 was intended to over-ride resistance caused by mutations. Crystallographic studies of AMN107 indeed suggest that subtle differences in the mode of binding to Abl and a better topological fit to the Abl protein account for the greater potency of the drug (Weisberg *et al*, 2005). Like imatinib, AMN107 binds to the inactive conformation of the Abl tyrosine kinase, with P-loop folding over the ATP-binding site, and the activation-loop blocking the substrate binding site, to disrupt the ATP-phosphate-binding site and inhibit the catalytic activity of the enzyme (Figure 1C) (Manley *et al*, 2005). AMN107 makes four hydrogen-bond interactions with the Abl kinase domain (Figure 1E), involving the pyridyl-N and the backbone-NH of Met318, the anilino-NH and the side-chain hydroxyl of Thr315, the amido-NH and side-chain carboxylate of Glu286, as well as the amido-C=O and backbone-NH of Asp381, to induce the inactive conformation of *BCR-ABL* (Figure 1C) (Manley *et al*, 2005). However, the many lipophilic interactions are also important for affinity, as is the interaction between the backbone-C=O of Asp381 and a fluorine atom in the trifluoromethyl group of AMN107 (Manley *et al*, 2005).

AMN107 is ⩾20-fold more potent than imatinib in the killing of wild-type *BCR-ABL*-expressing cells (Table 1) (Manley *et al*, 2005; O'Hare *et al*, 2005; Weisberg *et al*, 2005). Studies involving the imatinib-sensitive cell lines KBM5 and KBM7 show AMN107 to be 43- and 60 times more potent than imatinib, respectively (Golemovic *et al*, 2005). AMN107 maintains activity against 32/33 imatinib-resistant *BCR-ABL* mutants, but has no significant activity against the T315I mutant (Table 1) (Manley *et al*, 2005; O'Hare *et al*, 2005; Weisberg *et al*, 2005). As with imatinib, the lack of activity against the T315I mutant is the result of AMN107 binding closely to the T315 residue, such that loss of the hydroxyl side chain and additional methyl group of the isoleucine inhibits binding (Figure 1E and F).

In a dose-escalating Phase I study, imatinib-resistant CML patients in the chronic phase (17 patients), accelerated phase (46 patients) and blast crisis (33 patients), together with 13 Ph+ ALL patients, were treated with AMN107 (50–1200 mg day^−1^) for up to 385 days (Kantarjian *et al*, 2006). The maximum tolerated dose was determined to be 600 mg b.i.d., with frequently noted side effects being myelosuppression, mild-moderate skin rash and transient indirect hyperbilirubinaemia. In this study, AMN107 was not associated with the oedema frequently associated with imatinib. Among patients with chronic, accelerated and blast-phase CML, haematological/cytogenetic responses were achieved in 92/53, 72/48 and 39/27%, respectively. The best responses were seen at doses ⩾400 mg q.d. and with 400 mg b.i.d. Two of the imatinib-resistant Ph+ ALL patients also responded. Pharmacokinetic analysis of patients receiving 400 mg b.i.d., which was the dose selected for Phase II trials, showed mean peak-trough plasma levels of 3.6 and 1.7 *μ*M, respectively, with an apparent half-life of 15 h. Based upon the *in vitro* data, this level of drug exposure would be expected to result in clinical activity against most of the mutants characterised in Table 1, with the exception of T315I, and is therefore consistent with the responses observed in patients harbouring imatinib-resistant point mutations.

The early-phase clinical trials, therefore, support the possibility that AMN107 will have substantial clinical utility in rescuing patients who develop imatinib resistance due to point mutations, and could potentially be used as a single agent in patients at risk for progression. Additionally, there is growing interest in testing the hypothesis that administration of multiple Abl kinase inhibitors in early-phase patients, such as AMN107, dasatinib (Shah *et al*, 2005) and imatinib, could be used to delay or prevent the emergence of drug-resistant clones. In support of these ideas, additive/synergistic toxicity against both imatinib-sensitive and imatinib-resistant *BCR-ABL*-expressing cells has been reported following coadministration of AMN107 and imatinib, *in vitro* and *in vivo* (Griffin and Weisberg, 2005; Weisberg *et al*, 2005). Such effects might result from pharmacodynamic effects, and preliminary data suggest that synergy between imatinib and AMN107 may occur at the level of the CML stem cell due to the ability of both imatinib and AMN107 to inhibit or act as substrates of the multidrug efflux transporter ABCG2, which confers resistance toward several anticancer drugs (Jorgensen *et al*, 2005). A recent report also suggests that imatinib and AMN107 are taken up in cells by different mechanisms, with the influx, intracellular concentrations of imatinib, and consequently patient sensitivity to imatinib depending upon the organic cation transporter Oct-1, whereas AMN107 transport appears to be independent of Oct-1 (White *et al*, 2006). However, since the T315I mutation of *BCR-ABL* is highly resistant to imatinib, AMN107 and dasatinib, this approach needs to be extended to include inhibitors of T315I *BCR-ABL* to prevent this mutation from becoming more prevalent. Alternatively, it is also important to explore the potential for synergy between AMN107 and other classes of inhibitors that work through mechanisms not involving inhibition of Abl tyrosine kinase activity.

To aid the selection of patients most likely to benefit and show clinical responses to single agents, as well as to assess which drug combinations might be most appropriate, it is important to be able to predict resistance mechanisms and establish the resistance profiles of the available *BCR-ABL* inhibitors. Although overexpression of *BCR-ABL* is a possible resistance mechanism for AMN107 (Mahon *et al*, 2004), resistance is more likely to arise through the emergence of clones expressing AMN107-resistant mutant forms of *BCR-ABL*. A cell-based screening assay designed to predict such mutations has recently been applied to AMN107 (Von Bubnoff *et al*, 2006). Using this system, a reduced pattern of mutations was observed for AMN107, having some overlap with that seen for imatinib: Q252H, Y253H, E255K(V), F311I, T315I, S349L and F359I(V), all of which, with the exception of the T315I mutant, were suppressed at clinically achievable concentrations of AMN107. In an alternative cell-line-based mutagenesis study, the emergence of *BCR-ABL* mutations resistant to imatinib, AMN107 and dasatinib were compared: 18 mutations were recovered with imatinib, nine mutations (G250E, Y253H, E255K(V), E292V, T351I, F359C, L384M and L387F) were recovered with AMN107, and six mutations (E255K, L284V, V299L, T315I and F317I(V)) were recovered with dasatinib (Deininger *et al*, 2005). In a similar mutagenesis study with dasatinib (Shah *et al*, 2005), 10 resistance mutants of *BCR-ABL* involving six residues were isolated: L248R, Q252H, E255K, V299L, F317L/V/I/S and T315I/A. *BCR-ABL* point mutations conferring resistance to AMN107 have also been identified in a random mutagenesis study (Ray *et al*, 2005). In this study, 11 novel mutations were detected (K247N, L248V, L273F, E282K, K285N, V289L, E292K, N297T, H375P, T406I and W430L), in addition to five (Q252H, Y253C(H), E255K and T315I), which have been previously observed in CML patients treated with imatinib. Although these studies do not consistently identify the same drug-resistant *BCR-ABL* point mutations for individual drugs, it is clear that all three compounds display different mutagenicity profiles.

Since the pattern of arising *BCR-ABL* mutants should be associated with the binding mode of that particular compound to the Abl protein, conceptually, the greatest benefit from a combination of two such agents should be achieved using compounds having the greatest difference between their binding modes. Thus, whereas both imatinib and AMN107 bind to an inactive conformation of Abl (Figure 1C), dasatinib has been shown to bind to the active conformation (Figure 1A), and this can be invoked to explain the differences observed in the mutagenesis studies with these compounds. Therefore, a combination between dasatinib and AMN107 (or imatinib) might be expected to impart the greatest benefit (cf. Figure 1B), since dasatinib might inhibit many AMN107/imatinib-resistant mutants and conversely AMN107/imatinib might inhibit many dasatinib-resistant mutants.

Other studies have uncovered additional targets of AMN107 that help to elucidate its mechanism of action and/or suggest additional disease targets. Both AMN107 and imatinib have been observed to promote the expression of Bcl-2-interacting mediator, a tumour suppressor reported to be underexpressed in primary CML cells in comparison to normal cells (Aichberger *et al*, 2005). The ability of AMN107 to inhibit TEL-platelet-derived growth factor receptor-beta (TEL-PDGFRbeta), which causes chronic myelomonocytic leukaemia, and FIP1-like-1-PDGFRalpha, which causes hypereosinophilic syndrome, suggests potential use of AMN107 for myeloproliferative diseases characterised by these kinase fusions (Stover *et al*, 2005; Weisberg *et al*, 2005). AMN107 also inhibits the c-Kit receptor kinase, including the D816V-mutated variant of KIT, at pharmacologically achievable concentrations, supporting potential utility in the treatment of mastocytosis, and gastrointestinal stromal tumours (Weisberg *et al*, 2005; von Bubnoff *et al*, 2005; Gleixner *et al*, 2006).

## CONCLUSION

Preclinical and early-phase clinical findings indicate that AMN107 may be useful in the treatment of imatinib-refractory CML. This is due to its strong binding affinity to Abl, its activity against imatinib-resistant *BCR-ABL* point mutants, and its efficacy and tolerability in clinical studies. Greater than 70% of CML patients with advanced disease and over 90% of early, chronic-phase patients have responded to AMN107, and response rates continue to increase with overall good tolerability. In order to evaluate AMN107 in newly diagnosed CML, a study has recently been initiated (MD Anderson Cancer Center, Houston).

The failure of some patients to respond to AMN107, especially those with more advanced disease, might arise due to development of new mutations that impede the interaction between AMN107 and *BCR-ABL*. Thus, the identification and characterisation of *BCR-ABL* point mutants conferring resistance to AMN107 will assist in the prediction of patient responses to AMN107, identifying combination partners, as well as in the design and development of novel inhibitors of *BCR-ABL* that can over-ride resistance to such mutants. The preclinical and clinical evaluation of combinations of AMN107 with other approved or investigational inhibitors of Abl and additional signaling pathways will be helpful in the development of therapeutic strategies designed to over-ride drug resistance.

Both the safety and effectiveness of AMN107 are currently being evaluated in clinical trials involving CML patients that are intolerant of, or refractory to, imatinib. Thus far, AMN107 is showing promise as a potential therapeutic for CML at all levels of the disease. The frequency of use of AMN107 as a treatment for CML and Ph+ ALL will depend on its safety/efficacy profiles in clinical trials.

## Figures and Tables

**Table 1:** Comparison of imatinib and AMN107 for effects on autophosphorylation and proliferation in Ba/F3 cells transfected to express native *Bcr-Abl-* or imatinib-resistant mutant forms of the enzyme

	**Imatinib**		**AMN107**	
***Bcr-Abl* form (construct)**	**Autophosphorylation**	**Proliferation**	**Autophosphorylation**	**Proliferation**
Wild-type p210+IL-3	NA	>7700 (4)	NA	>10000 (15)
Wild-type p210	221±31 (14)	678±39 (23)	20±2 (7)	25±1 (68)
M237I (p185)	399 (2)	1545 (2)	41±8.3 (3)	43±8.7 (3)
M244V (p185)	937 (2)	2036 (2)	101±16 (3)	67±7 (4)
L248V (p185)	1011 (2)	2081 (2)	83±7 (3)	102±13 (4)
G250A (p185)	313 (2)	1269 (2)	58±11 (3)	65±5.6 (3)
G250E (p185)	2287±826 (4)	3329±1488 (2)	92±10 (5)	145±32 (3)
G250V (p185)	489 (2)	624 (2)	66±12 (3)	19±1.4 (3)
Q252H (p185)	1080±119 (2)	851±436 (2)	117±25 (3)	67±22 (4)
Y253H (p185)	>10000 (2)	>7000 (2)	260±34 (6)	700±116 (5)
E255D (p185)	754 (2)	1082 (2)	51±4.8 (3)	27±3.1 (3)
E255K (p185)	4856±482 (4)	5567 (2)	392±82 (6)	308±42 (5)
E255K (p210)	2455±433 (4)	7161±970 (3)	153±9 (4)	548±72 (6)
E255R (p185)	1877 (2)	1567 (2)	240±6.5 (3)	58±4.2 (3)
E255V (p210)	6353±636 (14)	6111±854 (12)	244±22 (13)	791±67 (19)
E275K (p185)	1038 (2)	563 (2)	125±5.0 (3)	44±17.1 (3)
D276G (p185)	1284 (2)	2486 (2)	107±9.1 (3)	69±10 (3)
E281K (p185)	584 (2)	1601 (2)	42±6.5 (3)	40±9.8 (3)
K285N (p185)	919 (2)	1264 (2)	204±19 (3)	57±12 (3)
E292K (p210)	275±81 (3)	1552 (2)	31±6 (3)	81±8 (4)
F311V (p185)	1480 (2)	3535 (2)	84±2 (3)	155±31 (4)
T315I (p210)	>10 000 (22)	>7000 (17)	>10 000 (48)	>10 000 (51)
F317C (p185)	1090 (2)	694 (2)	69±13 (3)	20±3.1 (3)
F317L (p210)	797±92 (11)	1528±227 (15)	38±4 (13)	91±6.5 (17)
F317V (p185)	544±47 (3)	549±173 (4)	95±28 (3)	28±4 (4)
D325N (p185)	584 (2)	887 (2)	70±9.0 (3)	26±2.7 (3)
S348L (p185)	553 (2)	1370 (2)	55±1.3 (3)	26±4.8 (3)
M351T (p210)	593±57 (11)	1682±233 (18)	29±3 (13)	38±4 (18)
E355A (p185)	676 (2)	1434 (2)	90±17 (3)	35±6.7 (3)
E355G (p185)	601 (2)	1149 (2)	67±15 (3)	47±8 (4)
F359C (p185)	1130 (2)	2377 (2)	217±17 (3)	258±61 (3)
F359V (p185)	1528 (2)	595 (2)	313±79 (3)	161±61 (4)
A380S (p185)	2617 (2)	3744 (2)	135±11 (3)	164±27 (3)
L387F (p185)	530 (2)	172 (2)	197±25 (3)	46±7.2 (3
M388L (p185)	517 (2)	525 (2)	73±16 (3)	18±2.6 (3)
F486S (p210)	1238±110 (11)	3050±597 (10)	41±4 (8)	75±7 (11)

The influence of compounds on kinase autophosphorylation or cell viability was calculated as percentage inhibition as described (Weisberg *et al*, 2005). Dose–response curves were used to calculate IC_50_ values, expressed as mean±s.e.m. (nM) (number of replicates). The influence of compounds on *Bcr-Abl* autophosphorylation or cell viability was determined with capture ELISAs or the ATPlite™ assay kit (Perkin-Elmer), respectively. Dose–response curves (per cent inhibition) were used to calculate IC_50_ values, expressed as mean±s.e.m., *n*=number of experiments.
