# Correction: AMN107 (nilotinib): a novel and selective inhibitor of *BCR-ABL*

**DOI:** 10.1038/s41416-019-0505-7

**Published:** 2019-06-19

**Authors:** E Weisberg, P Manley, J Mestan, S Cowan-Jacob, A Ray, J D Griffin

**Affiliations:** 1 Department of Adult Oncology, Dana Farber Cancer Institute, 44 Binney Street, Boston, MA 02115 USA; 2 Novartis Institutes of Biomedical Research, Basel, Switzerland

**Correction to**: *British Journal of Cancer* (2016) **94**, 1765-1769; 10.1038/sj.bjc.6603170; published online 23 May 2006.

Since the publication of this paper, the authors have reported that there is an error in the chemical structure for imatinib presented in Fig. 1f. The correct version of Fig. 1 with chemical structure is provided below.

**Fig. 1:**
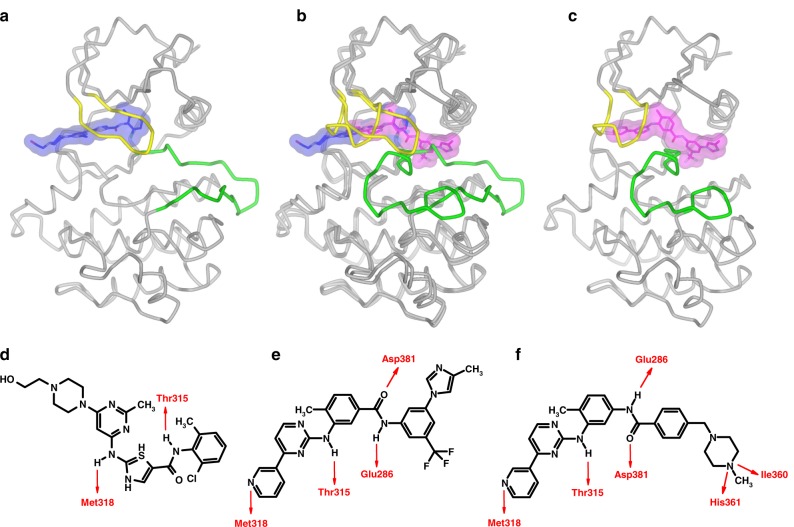
Structures of Abl kinase (**a**) in the active (Fendrich et al., 2006) and (**c**) inactive states, with dasatinib (blue) docked and nilotinib (magenta) as bound in the crystal structure (Weisberg et al., 2005), respectively. The differing conformations of the glycine-rich or P-loop (yellow) and the activation loop (green) are induced or stabilised by the different binding modes of the two inhibitors. **b** shows a superposition of the two distinct conformations, emphasising how dasatinib and nilotinib occupy different parts of the cleft between the N- (upper) and C-terminal (lower) lobes of the kinase. The corresponding aspects of the molecular structures of (**d**) dasatinib and (**e**) nilotinib are depicted, with their respective H-bond interactions with the Abl kinase domain indicated in red, in comparison to imatinib (**f**)

